# The Use of Animations Depicting Cardiac Electrical Activity to Improve Confidence in Understanding of Cardiac Pathology and Electrocardiography Traces Among Final-Year Medical Students: Nonrandomized Controlled Trial

**DOI:** 10.2196/46507

**Published:** 2024-04-23

**Authors:** Alexandra M Cardoso Pinto, Daniella Soussi, Subaan Qasim, Aleksandra Dunin-Borkowska, Thiara Rupasinghe, Nicholas Ubhi, Lasith Ranasinghe

**Affiliations:** 1 School of Medicine, Imperial College London, London, United Kingdom; 2 School of Medicine, University College London, London, United Kingdom; 3 University Hospitals Sussex NHS Foundation Trust, Sussex, United Kingdom; 4 Imperial College Healthcare NHS Trust, London, United Kingdom

**Keywords:** medical education, cardiology, technology, clinical skills, cardiac, cardiac electrical activity, ECG, mixed methods study, students, education, medical professionals, development, web-based tutorial, teaching, cardiovascular, learning, electrocardiography

## Abstract

**Background:**

Electrocardiography (ECG) interpretation is a fundamental skill for medical students and practicing medical professionals. Recognizing ECG pathologies promptly allows for quick intervention, especially in acute settings where urgent care is needed. However, many medical students find ECG interpretation and understanding of the underlying pathology challenging, with teaching methods varying greatly.

**Objective:**

This study involved the development of novel animations demonstrating the passage of electrical activity for well-described cardiac pathologies and showcased them alongside the corresponding live ECG traces during a web-based tutorial for final-year medical students. We aimed to assess whether the animations improved medical students’ confidence in visualizing cardiac electrical activity and ECG interpretation, compared to standard ECG teaching methods.

**Methods:**

Final-year medical students at Imperial College London attended a web-based tutorial demonstrating the 7 animations depicting cardiac electrical activity and the corresponding ECG trace. Another tutorial without the animations was held to act as a control. Students completed a questionnaire assessing their confidence in interpreting ECGs and visualizing cardiovascular electrical transmission before and after the tutorial. Intervention-arm participants were also invited to a web-based focus group to explore their experiences of past ECG teaching and the tutorial, particularly on aspects they found helpful and what could be further improved in the tutorial and animations. Wilcoxon signed-rank tests and Mann-Whitney *U* tests were used to assess the statistical significance of any changes in confidence. Focus group transcripts were analyzed using inductive thematic analysis.

**Results:**

Overall, 19 students attended the intervention arm, with 15 (79%) completing both the pre- and posttutorial questionnaires and 15 (79%) participating in focus groups, whereas 14 students attended the control arm, with 13 (93%) completing both questionnaires. Median confidence in interpreting ECGs in the intervention arm increased after the tutorial (2, IQR 1.5-3.0 vs 3, IQR 3-4.5; *P*<.001). Improvement was seen in both confidence in reviewing or diagnosing cardiac rhythms and the visualization of cardiac electrical activity. However, there was no significant difference between the intervention and control arms, for all pathologies (all *P*>.05). The main themes from the thematic analysis were that ECGs are a complex topic and past ECG teaching has focused on memorizing traces; the visualizations enabled deeper understanding of cardiac pathology; and ECG learning requires repetition, and clinical links remain essential.

**Conclusions:**

This study highlights the value of providing concise explanations of the meaning and pathophysiology behind ECG traces, both visually and verbally. ECG teaching that incorporates relevant pathophysiology, alongside vignettes with discussions regarding investigations and management options, is likely more helpful to students than practices based solely on pattern recognition. Although the animations supported student learning, the key element was the tutor’s explanations. These animations may be more helpful as a supplement to teaching, for instance, as open-access videos.

## Introduction

Electrocardiography (ECG) interpretation is a fundamental skill necessary during medical school education and in the practice of clinical medicine and surgery. Recognizing pathologies such as an ST elevation or non-ST elevation myocardial infarction; bundle branch block (BBB); and arrhythmias, including atrial fibrillation (AF), supraventricular tachycardia, ventricular fibrillation, and ventricular tachycardia, allows for prompt intervention and improves patient care, especially in the acute setting where urgent interventions may be lifesaving.

Despite its importance, medical students struggle with interpreting ECGs, and teaching methods seem to vary greatly between systematic interpretation based on ECG segments and pattern recognition. This leads to a lack of confidence and inaccurate interpretation of ECGs, which could lead to adverse events including treatment delay or incorrect management of the pathology. Although automated computer interpretation may be available, this should not be used by itself to diagnose conditions, since clinical correlation is warranted and these algorithms may be inaccurate [1].

The major disruptions caused by the COVID-19, in which medical students had limited exposure to hospital wards and experienced most lectures and tutorials on the web rather than in person [2], served as a strong reminder of the need for investment in innovative teaching methods.

Literature already suggests that teaching should be focused on the understanding of lead placement, as well as the basics of electrophysiology and ECG, to better identify abnormalities [3]. Teaching should also be correlated with the clinical findings of a case, as this has been shown to lead to more accurate ECG diagnosis in practice. For instance, case-based learning (CBL) has been frequently used in recent years, which increases the practical knowledge and confidence of medical students and junior doctors, through clinically correlating various cardiac pathologies [4]. However, explaining the link between the underlying cardiac pathology and the traces demonstrated by the ECG is not common practice in medical school curricula.

Medical students repeatedly describe ECG interpretation as a challenging skill [5,6]. A study based in Israel reports that despite competence and confidence in ECG interpretation improving throughout medical school, levels remain low among final-year medical students [5]. Moreover, a study of Polish medical students highlighted students’ lack of ability to recognize common and emergency cardiac pathologies [6]. Additionally, these results emphasized independent learning as the strongest predictor of competency, as opposed to attendance in formal teaching sessions [6]. This continues following medical school, with rates of accurate ECG interpretation being as low as 55.8% among trainee doctors [7]. These findings suggest the need for a review of ECG teaching methods.

Technology-enhanced learning has grown in popularity and has been trialed as a method to encourage active practice of ECG interpretation among medical students. Students in this cohort demonstrated better diagnostic accuracy, but rates of knowledge attrition 6 months after the study remained high [1]. These findings highlight that despite continued practice remaining important, current methods of teaching ECGs do not support students in gaining in-depth understanding, nor do they enable knowledge retention.

Methods of technology-enhanced education, including visualizations, have been trialed extensively for anatomy teaching [8], with reported increases in student engagement with the content [9]. Although greater engagement does not ensure improved understanding, it may be an important component in supporting effective teaching and learning [8]. Additionally, there is evidence to show that visualization tools are capable of supporting students’ understanding of anatomy [10].

Therefore, this research team developed novel animations demonstrating the passage of electrical activity through the heart for different pathologies and showcased them alongside the corresponding live ECG traces during a web-based ECG tutorial for final-year medical students at Imperial College London. The aim of this study was to assess whether these animations are associated with the improvement of final-year medical students’ confidence in both visualizing cardiac pathology and interpreting the corresponding ECGs, compared to standard ECG teaching methods that do not involve visual animations.

## Methods

The study was designed as a nonrandomized controlled trial.

### Recruitment

Year 6 Bachelor of Medicine, Bachelor of Surgery students from Imperial College London were invited to participate in the study. Messages were sent through student communication channels with the description of the study and tutorial. This included a link to the study information sheet as well as a sign-up link to register their interest in participating. The first 20 students to sign up were emailed by a member of the research team with the information sheet and focus group consent form attached. Students were asked to confirm their participation by returning the signed consent form via email. Students were given a week to confirm their participation, after which the space would be offered to others who registered interest until a total of 20 confirmations were reached. The process was repeated in the following year, with a new cohort of Year 6 Bachelor of Medicine, Bachelor of Surgery students at the same point in the academic year as the original cohort.

The sample size of 20 students per teaching session was agreed by the research team based on the tutor’s preference, following their experience of what would be a feasible number of students to teach within the agreed timeframe. This decision was also supplemented by evidence to suggest that cohorts of fewer than 30 students may enable better learning [11] and that cohorts of approximately 19 students may enable greater interaction [12].

### Design and Delivery of the Tutorial

Prototypes for the ECG traces and animations were created on Microsoft PowerPoint by 2 junior doctors on the research team. A total of 7 ECG patterns (sinus rhythm, AF, atrial flutter, atrioventricular nodal re-entry tachycardia, atrioventricular re-entry tachycardia, right BBB, and left BBB) that are known to commonly arise in clinical practice and in exams were chosen, and the prototypes were converted into high production value animations using Adobe Illustrator and Adobe After Effects. The animations were produced by skilled members of the research team and took a collective total of 10 hours to produce. The final product consisted of a video animation of the electrical activity passing through the heart alongside an ECG rhythm strip (lead II) for the given abnormality—with the exception of BBBs, which were depicted alongside leads V1 and V6. The animation of electrical activity through the heart and the corresponding ECG trace were synchronized to demonstrate how each ECG deflection corresponds with the electrical activity within the heart. Depolarization was shown in yellow and repolarization was shown in green.

The tutorials were both delivered by a UK-based Academic Foundation doctor within the research team (LR) on Zoom (Zoom Video Communications) at a prespecified time on a weekday evening. LR has vast experience teaching medical students and designing medical educational material and had completed the Membership of the Royal Colleges of Physicians of the United Kingdom Part 1 Examination successfully at the time of delivering the sessions.

Participants logged on using their unique identifier code and kept their cameras turned off to maintain anonymity. The intervention tutorial involved going through each animation in turn and narrating the path of the electrical activity. To ensure the smooth running of the event, questions were reserved until the end of the session.

The control tutorial followed the same lesson plan as the intervention but involved the tutor narrating the path of electrical activity using an example 12-lead ECG without any animations. Participants were not explicitly told this tutorial would be the control arm but were instead invited to a standard ECG tutorial, following the same methods as the intervention. However, all participants would have read the study information sheet and known the aim of the study, which may have compromised the single-blinding process.

### Questionnaires

An email was sent to participants 1 week prior to the tutorial with a link to an anonymous Qualtrics questionnaire to be completed before the tutorial (Multimedia Appendix 1). This questionnaire was composed of 5-point Likert-scale questions assessing participants’ confidence in interpreting ECGs and visualizing cardiovascular electrical transmission in each of the cardiovascular pathologies covered in the tutorial. The questionnaire also included multiple-choice and free-text questions inquiring about previous formats of ECG teaching experienced by participants and their views on what could be improved about current ECG teaching generally.

A similar questionnaire was repeated at the end of the tutorial to assess change in confidence using the same 5-point Likert-scale questions, as well as free-text questions inquiring about participants’ experience of the tutorial (Multimedia Appendix 1). A link and QR code to this questionnaire was shared at the end of the tutorial, prior to the start of the focus groups.

Participants were given a unique participant code, which they were asked to state at the start of each questionnaire. This enabled questionnaire responses to be paired while maintaining anonymity.

### Focus Groups

Focus groups were only conducted at the end of the intervention tutorial. Participants who took part in the control arm were not invited for a focus group, as the primary purpose of this exercise was to understand participants’ experience of the visual animations, which were not included in the control arm. Upon the completion of the tutorial, students were divided into 4 breakout rooms on Zoom, each designed to host 5 students and a single researcher. Participants were asked to unmute microphones to participate in the semistructured focus group and were invited to keep their cameras switched off if they wished to remain anonymous. The focus groups further explored participants’ experiences of past ECG teaching and the current tutorial, with particular focus on aspects they found helpful and what could be further improved in the delivery of the tutorial and design of the visualizations (Multimedia Appendix 2).

Focus group questions were designed collaboratively by the research team, with feedback from an expert qualitative researcher (see the *Acknowledgments* section), to ensure that the questions were adequate in informing the study’s aims and gave participants the opportunity to share their experiences of ECG learning openly. These were also reviewed by the ethics committee (see the *Ethical Considerations* section).

Focus groups were audio and video recorded on the platform. Recordings were deleted upon transcription, which took place within 2 weeks of the tutorial. Participants were asked if they wished to receive a copy of the transcription to review their statements (anonymized using their unique participant codes); those who asked for the transcription were sent the transcript by email and given 1 week to inform the research team of any redactions they wished to make.

### Data Analysis

Questionnaire data were analyzed using descriptive statistics on Microsoft Excel. The Shapiro-Wilk test was used to determine the distribution of data. As this showed that the data were nonparametric, Wilcoxon signed-rank tests were used to assess the statistical significance of any reported changes in confidence between pre- and posttutorial questionnaire responses, for each of the intervention and control arms. The Mann-Whitney *U* test was used to compare differences in pre- and posttutorial confidence between the intervention and control arms of the study.

Focus group transcripts were analyzed using inductive thematic analysis, following Braun and Clarke’s [13] stages of thematic analysis as guidance. This was done on NVivo 12.0 (Lumivero) by 2 researchers cooperatively. Themes were reviewed by a third researcher. Free-text questions from the questionnaires were analyzed following similar methods on Microsoft Excel.

### Ethical Considerations

This study was approved by the Imperial College Education Ethics Review committee (EERP2122-086). Participation in questionnaires and focus groups was voluntary, with participants given the option to withdraw from the study at any point, up until 2 weeks following the completion of the postintervention questionnaire. All participants were provided with a study information sheet prior to confirming their consent for participation in the study. There was no financial compensation involved in this study. Information sheets explained the aim of the study, methods of data storage, and outputs. Participants were also provided with a unique identifier code generated by the research team to be placed at the start of the questionnaires, enabling data to remain paired while ensuring anonymity.

## Results

### Questionnaire Results

The first 20 students who signed up to participate in each tutorial were allocated a slot.

In the intervention tutorial, a total of 19 students attended. Of these, 15 (79%) completed both the pre- and posttutorial questionnaires.

All participants confirmed at least 1 prior method of ECG teaching, including didactic lectures (13/15, 87%), case-based tutorials (11/15, 73%), memorization of ECG features (9/15, 60%), animations (2/15, 13.3%), and practical sessions (1/15, 7%).

**Figure 1.**
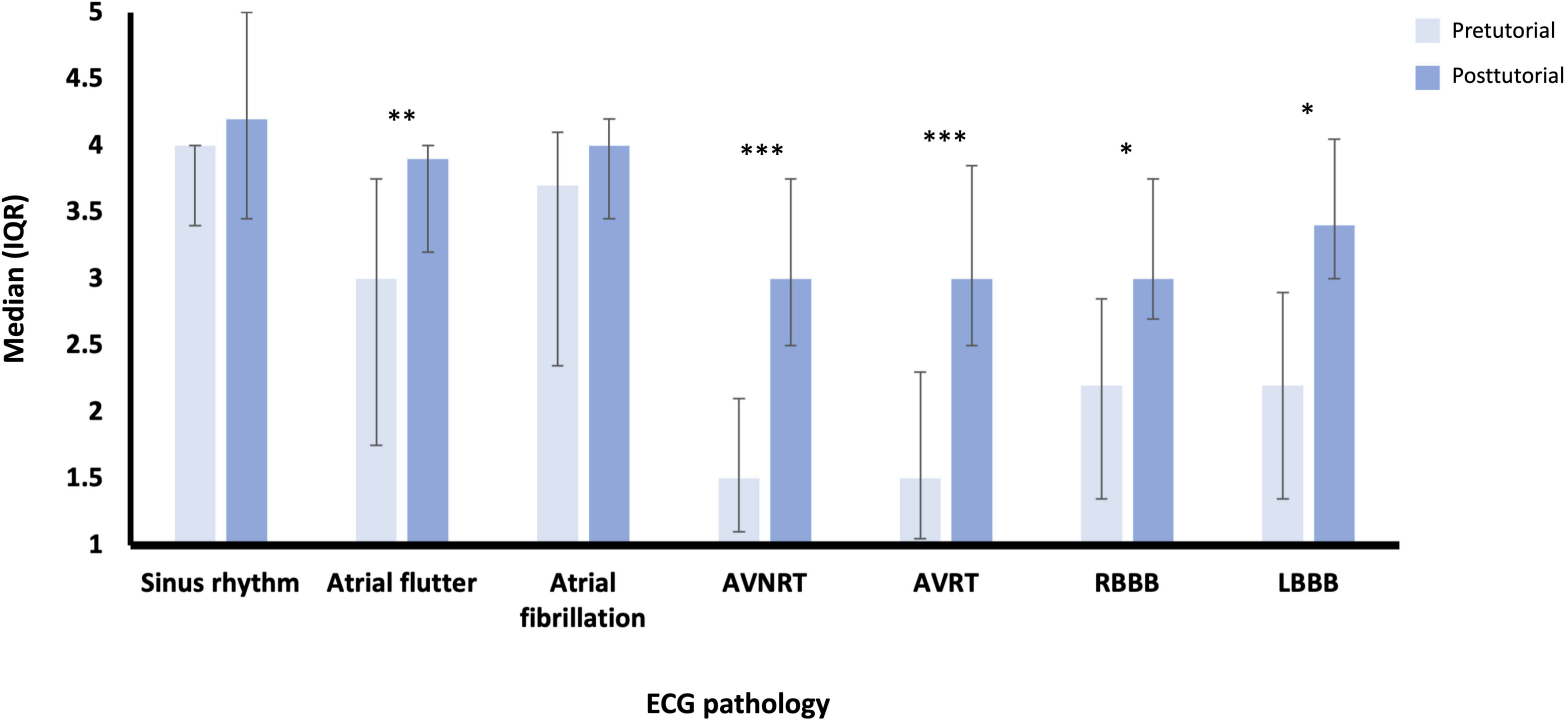
Confidence in reviewing and diagnosing cardiac rhythms and pathology on an ECG (median and IQR score on a Likert scale, from a 1=not confident at all to 5=extremely confident) for the intervention group (n=15). Wilcoxon signed-rank test results: *P≤.05, **P≤.01, and ***P≤.001. AVNRT: atrioventricular nodal re-entry tachycardia; AVRT: atrioventricular re-entry tachycardia; ECG: electrocardiography; LBBB: left bundle branch block; RBBB: right bundle branch block.

**Figure 2.**
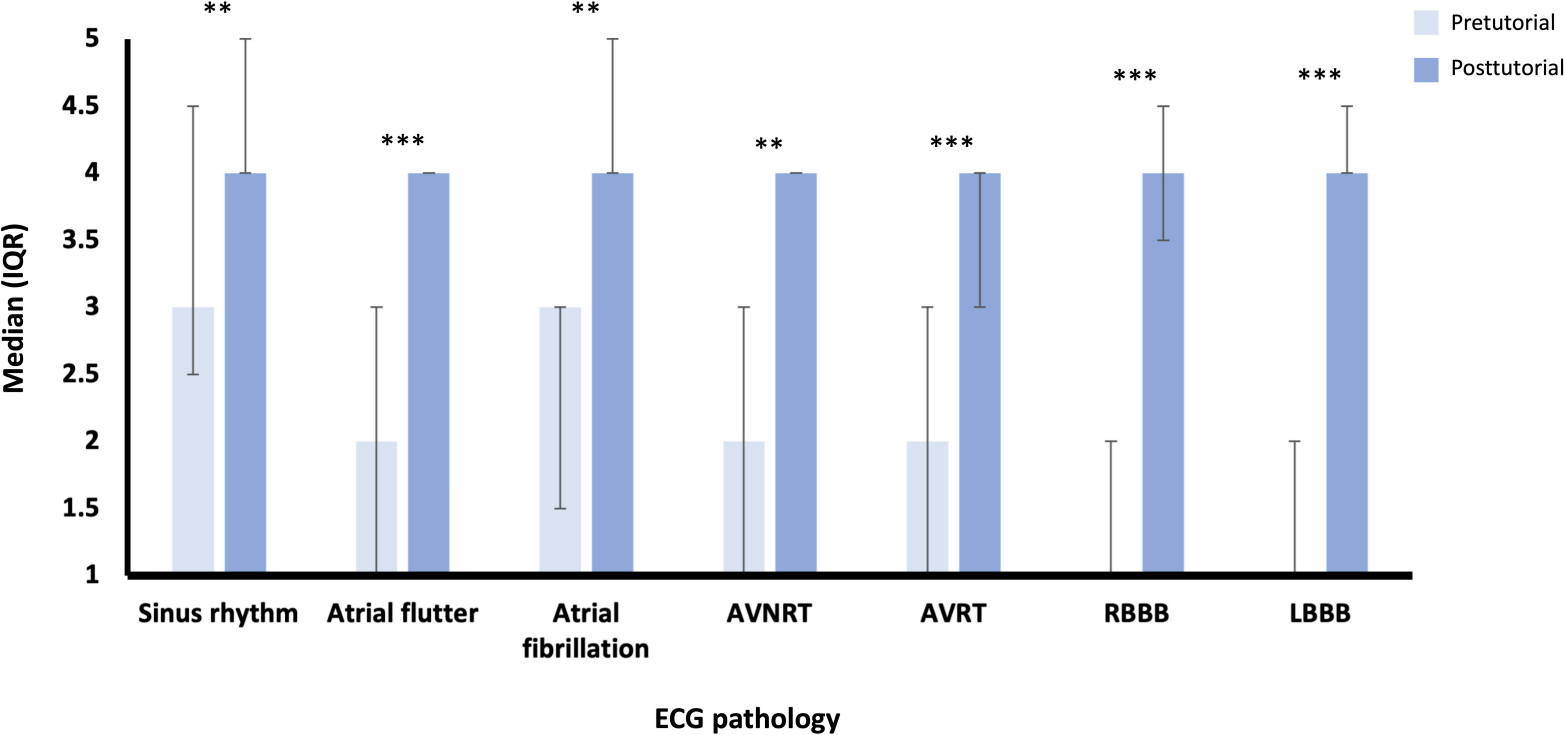
Confidence in visualizing electrical activity through the heart in different ECG pathologies (median and IQR score on a Likert scale, from a 1=not confident at all to 5=extremely confident) for the intervention group (n=15). Wilcoxon signed-rank test results: *P≤.05, ** *P≤*.01, and *** *P≤*.001. AVNRT: atrioventricular nodal re-entry tachycardia; AVRT: atrioventricular re-entry tachycardia; ECG: electrocardiography; LBBB: left bundle branch block; RBBB: right bundle branch block.

Overall, in the intervention group, median results for confidence in interpreting ECGs increased from the pretutorial scores (2, IQR 1.5-3) to the posttutorial scores (3, IQR 3-4.5; *P<*.001). Improvement was seen in both confidence in reviewing and diagnosing cardiac rhythms (Figure 1) and in visualizing electrical activity throughout the heart (Figure 2), across most of the pathologies illustrated.

Participants showed the least confidence in reviewing and diagnosing ventricular pathologies compared to atrial pathologies, with atrioventricular nodal re-entry tachycardia and atrioventricular re-entry tachycardia scoring the lowest median confidence scores before and after the tutorial but also showing the greatest levels of improvement following the tutorial (Figure 1; Table S1 in Multimedia Appendix 3).

A similar pattern is observed for median scores in visualizing cardiac electrical activity (Figure 2; Table S2 in Multimedia Appendix 3). However, median confidence levels before the tutorial in this category were, overall, lower than the same measurement of confidence for reviewing and diagnosing cardiac rhythms. Nevertheless, the level improvement in confidence in visualizing electrical activity in the heart was overall greater than that in confidence in reviewing and diagnosing cardiac rhythms, with median posttutorial confidence levels also achieving higher levels in most cardiac pathologies than those for reviewing and diagnosing cardiac rhythms.

Participants reported greater enjoyment of this tutorial (median 4, IQR 3-4.5) compared to past ECG teaching (median 3, IQR 1.5-3; *P*=.02).

For the control arm of the study, a total of 14 students attended the tutorial, of which 13 (93%) completed both the pre- and posttutorial questionnaires.

Prior methods of ECG teaching were similar to those of the intervention group, including didactic lectures (11/13, 85%), case-based tutorials (10/13, 77%), memorization of ECG features (6/13, 46%), practical sessions (3/13, 23%), animations (2/13, 15%), and a website with example ECGs for self-learning (1/13, 8%).

Overall confidence in interpreting ECGs showed only slight improvement in the control group, from a median of 3 (IQR 2-3) to 3 (IQR 3-4; *P*=.01).

Pretutorial confidence scores were similar in the control and intervention arms (Table 1). For the control group, pretutorial median confidence scores were also lower for ventricular pathologies compared to atrial pathologies, and overall confidence scores for reviewing and diagnosing cardiac pathologies were higher than visualizing cardiac activity, which is similar to the pattern seen in the intervention group (Multimedia Appendix 4).

**Table 1.** Pretutorial median (IQR) confidence scores for control and intervention groups with *P* values (Mann-Whitney *U* Test).

Scores and pathologies		Intervention, median (IQR)	Control, median (IQR)	*P* value
**Confidence in reviewing and diagnosing cardiac rhythms and pathology on an ECG^a^**				
	Sinus rhythm	4.0 (3.4-4.0)	5.0 (3.9-5.0)	.07
	Atrial flutter	3.0 (1.8-3.8)	3.3 (3.9-5.0)	.35
	Atrial fibrillation	3.7 (2.4-4.1)	4.0 (3.0-4.0)	.19
	AVNRT^b^	1.5 (1.1-2.1)	2.0 (1.3-2.5)	.32
	AVRT^c^	1.5 (1.1-2.3)	2.0 (1.5-2.5)	.39
	RBBB^d^	2.2 (1.4-2.9)	2.5 (2.0-3.5)	.28
	LBBB^e^	2.2 (1.4-2.9)	2.5 (2.0-3.5)	.33
**Confidence in visualizing electrical activity on an ECG**				
	Sinus rhythm	3.0 (2.5-4.5)	4.0 (2.0-4.0)	.59
	Atrial flutter	2.0 (1.0-3.0)	2.0 (1.0-2.0)	>.99
	Atrial fibrillation	3.0 (1.5-3.0)	2.0 (1.0-4.0)	.98
	AVNRT	2.0 (1.0-3.0)	2.0 (1.0-3.0)	.94
	AVRT	2.0 (1.0-3.0)	2.0 (1.0-2.0)	.68
	RBBB	1.0 (1.0-2.0)	2.0 (1.0-4.0)	.18
	LBBB	1.0 (1.0-2.0)	2.0 (1.0-4.0)	.18
**Overall confidence in interpreting ECGs**		2.0 (1.0-3.0)	3.0 (2.0-3.0)	.50

a ECG: electrocardiography.

b AVNRT: atrioventricular nodal re-entry tachycardia.

c AVRT: atrioventricular re-entry tachycardia.

d RBBB: right bundle branch block.

e LBBB: left bundle branch block.

There was no statistically significant difference between the enjoyment of this tutorial (median 4, IQR 4-5) compared to past ECG teaching (median 4, IQR 3-4; *P*=.052).

When comparing the change in confidence between the control and intervention groups for both reviewing and diagnosing pathology and visualizing electrical activity, no statistically significant difference was seen across all pathologies (all *P*>.05).

**Figure 3.**
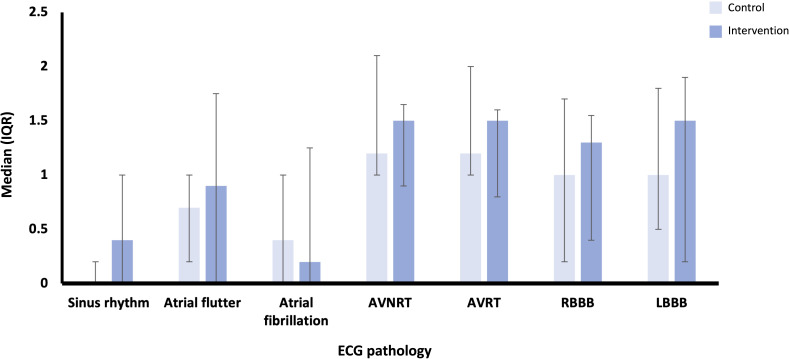
Median (IQR) scores for absolute difference in confidence in reviewing and diagnosing cardiac rhythms and pathology on an ECG for control (n=13) and intervention (n=15) groups. AVNRT: atrioventricular nodal re-entry tachycardia; AVRT: atrioventricular re-entry tachycardia; ECG: electrocardiography; LBBB: left bundle branch block; RBBB: right bundle branch block.

**Figure 4.**
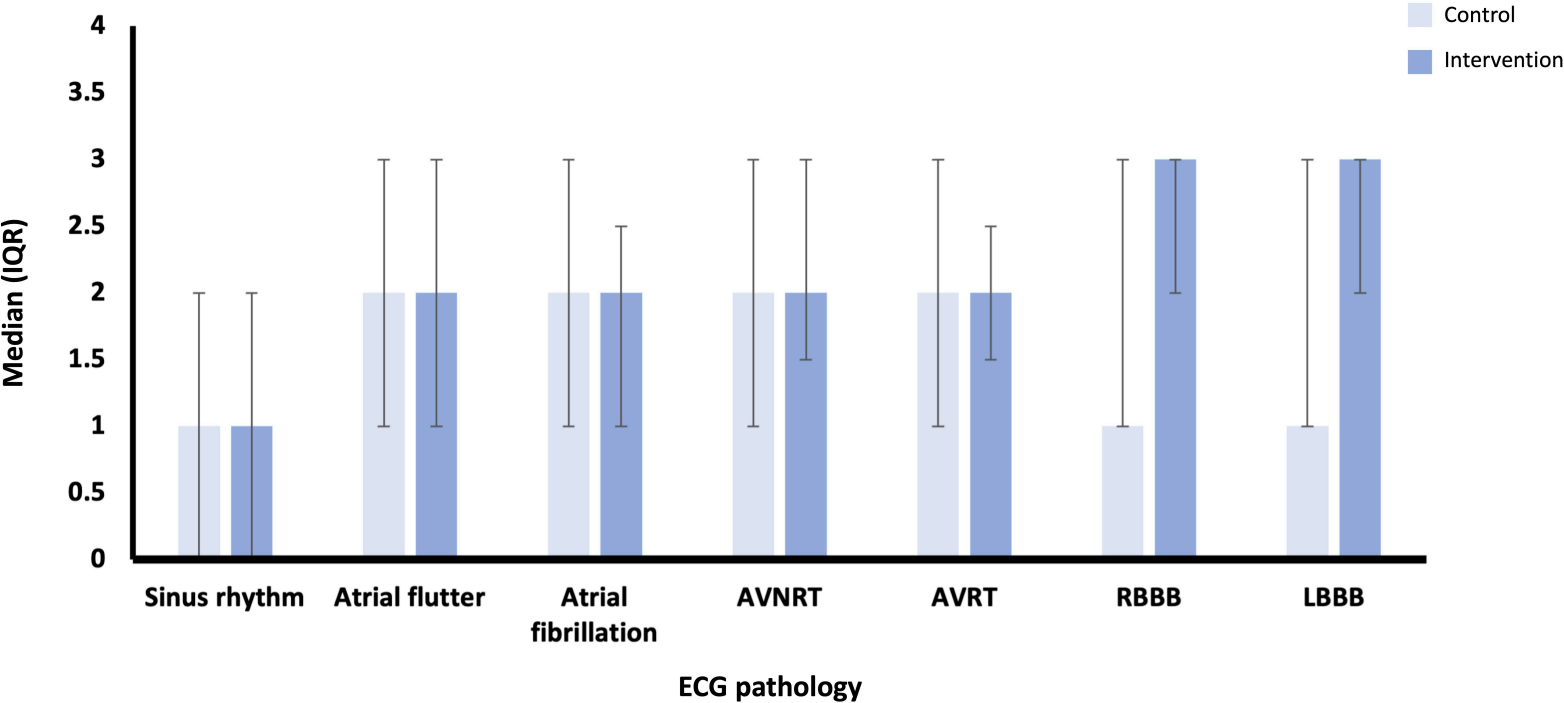
Median (IQR) scores for absolute difference in confidence in visualizing electrical activity through the heart in different ECG pathologies for control (n=13) and intervention (n=15) groups. AVNRT: atrioventricular nodal re-entry tachycardia; AVRT: atrioventricular re-entry tachycardia; ECG: electrocardiography; LBBB: left bundle branch block; RBBB: right bundle branch block.

Data for confidence in reviewing and diagnosing cardiac rhythms and pathology showed greater improvements in the intervention group across most pathologies, except for AF. The greatest absolute difference between the intervention and control groups was seen for left BBB, although this was still statistically nonsignificant (*P*=.89; Figure 3). Data for confidence in visualizing cardiac electrical activity showed similar median changes in confidence across most pathologies, apart from right and left BBBs, where the intervention group showed greater improvement, although not statistically significant (*P*=.15 and *P*=.12, respectively; Figure 4).

There was also no statistically significant difference in median scores for the enjoyment of the tutorial when comparing control and intervention groups (*P*=.37).

### Focus Group Results

#### Overview

A total of 15 (79%) out of 19 participants who attended the intervention tutorial took part in the focus groups. These were preallocated at random into 4 separate groups, which contained between 2 to 5 students (1 with 5 students, 2 with 4 students, and 1 with 2 students). Three key themes emerged from the analysis of focus group transcripts.

#### Past ECG Learning Has Been Centered on the Clinical Context and Memorizing Traces

All participants noted varied past ECG teaching, including formal lectures and tutorials focused on the principles of ECG interpretation throughout medical school but also informal teaching while on placement. However, there was an agreement that ECGs remained a challenging concept to learn. For example, one participant noted that they “found [ECGs] hard to understand and engage [with]” (participant 8), whereas another explained how “an ECG can kind of be a different language almost” (participant 7) and hence may take more time and effort to understand.

Past teaching experienced by participants, particularly informal teaching on placement, also focused on pattern recognition and correlating ECG signs to diagnoses.

*I think at least with the ones I went through with the doctors and stuff, it was very much like a tick box or like oh the saw tooth pattern is this, this is this…*

[Participant 12]

Although this format of learning was concise and focused on key knowledge required to be a Foundation Year 1 doctor, it did not promote deeper understanding of ECGs that could be applied to any ECG pattern.

*I’d kind of leave knowing that if that exact ECG comes up, that was helpful, but otherwise I don’t know really what or why it is that and then some of the actual understanding came from doing work outside of firms.*

[Participant 30]

Participants also highlighted that “the key thing is kind of just repetition” (participant 7) when learning to interpret ECGs, and that “you also have to dedicate time yourself to go through it, if you really want to properly understand it” (participant 33).

Being taught systematic methods for ECG interpretation and presentation was reported to be useful; namely, it was “more relevant to us and our exams and practicals” (participant 15) when going through clinical cases alongside ECGs, which help provide clinical context to the ECG and “also gets you used to different subtleties, because between patients an ECG of the same condition can look slightly different” (participant 6).

#### The Animations and Associated Explanations Promoted a Deeper Understanding of Cardiac Electrical Activity

Overall, participants found the animations and accompanying explanations during the tutorial to be a helpful tool. The depiction of an ECG trace and heart animation simultaneously helped them understand the correlation between the 2, and hence, as a participant stated, “the first time I’ve properly understood what is exactly is going on [in the heart]” (participant 1) for the pathologies illustrated. One participant highlighted that “breaking it down into basics…and how it’s reflected in the heart as well as it’s corresponding trace…feels less like I’m trying to memorise something and more like I’m actually trying to figure it out” (participant 17).

The visual nature of these animations enabled participants to “clearly see how the electricity is conducted in the heart” (participant 2) and was noted to be an effective method to “consolidate what I know about the conditions that we went through” (participant 2).

This more thorough level of understanding was noted to be “quite useful” to participants, as “when going on the wards and I see an ECG, I can actually visualise how the heart is functioning” (participant 17). It was also perceived to be a helpful way of retaining their learning about heart pathologies and associated ECG traces in the longer-term, as “when you understand the reason why something is the way it is you are more likely to remember it” (participant 15).

Participants also stated the value of covering content that they considered relevant to their exams and starting work as Foundation Year 1 doctors: “I enjoyed the fact that we covered like a lot of a main conditions, so less of the more niche stuff” (participant 7). They also described this tool as more of a helpful “recap” (participant 2) of heart conditions and their associated ECG traces, as opposed to methods of ECG interpretation, which are often the focus in later years of medical school. One participant explained, “I just wish we were taught this way before [in earlier years of medical school]; it would make understanding a lot easier later” (participant 12).

#### Implementing This 1-Hour Tutorial Is Not Enough: ECG Learning Requires Repetition and Clinical Links Remain Essential

The key differentiating component of this tutorial was its animations: *“*I’m a visual learner, so I need to see it to understand it. So that’s what’s been a gamechanger for me, to actually see the animation” (participant 32). However, participants suggested that “having [the animation playing] even slower” (participant 8) or the opportunity to independently “use the scroller to advance” (participant 32) through the animation would be helpful to visualize more carefully “what is happening step-by-step in the heart and on the ECG” (participant 32). One participant also suggested potential value in “3D animations, that would be useful so you can turn the heart around and see all the fibres and all the [conduction activity]” (participant 17).

Despite the value of the tutorial in supporting students’ understanding of cardiac pathologies, participants highlighted that there are additional factors that are important in contributing to in-depth learning. For instance, the need for repetition was widely acknowledged. Participants therefore asked that animations be made available for them to view independently. Additionally, the fast-paced nature of the tutorial, which covered multiple pathologies, means that some participants “didn’t really have much of a time to get an understanding again, of like the condition” (participant 7), which might be resolved through independent revision with the animations or delivering the content through multiple teaching sessions.

Finally, participants noted the value of greater interaction with the audience, including the implementation of quizzes to test understanding and the integration of clinical cases for stronger clinical correlations.

## Discussion

Overall, results for the intervention cohort demonstrate a statistically significant improvement in confidence when identifying abnormalities in ECG traces and visualizing cardiac electrical activity, compared to prior to attending this tutorial. However, a similar improvement was seen in the control group, with no statistically significant differences in improvement in confidence between the control and intervention groups. Although the focus groups highlighted a possible value in the use of animations demonstrating cardiac electrical activity synchronized to the corresponding ECG trace, the overall results suggest that perhaps this tool may be more adequate as a supplement to teaching.

Focus group transcripts provided fruitful data on how students have previously been taught how to interpret ECGs, how their previous learning compared to how this tutorial was delivered, and what they thought of the animations used to support the tutorial delivery. Moreover, information on how to improve the session was also collected. The main themes that arose were that ECGs are regarded as a complex topic among students and that past ECG learning used CBL and involved the memorization of traces. Other main themes include that the animations and associated explanations promoted a deeper understanding of cardiac electrical activity (compared to past teaching) and that ECG learning requires repetition and clinical links remain essential. Students noted that their most helpful past teaching involved cases and clinical contextualization, which should therefore be considered in any form of teaching implemented to final-year students, as clinical context appears to be their learning priority.

The key commonality between the control and intervention groups was the provision of a concise explanation of cardiac electrical activity in the heart for each section of the ECG trace. Therefore, future studies may benefit from investigating ways of delivering this content most effectively, for example through CBL or team-based learning [14-16], or similar methods of enabling greater interaction between students, but with a focus on understanding the pathology as opposed to focusing on pattern recognition.

This study and its teaching session do not come without some limitations. For reasons described in the *Methods* section, this study was limited to up to 20 participants in each arm and was based in a single study-year group and university. Therefore, it is not possible to confirm that these results are generalizable. No data were collected on the demographics of participants, which would also be helpful in determining the generalizability of the findings. Furthermore, this study also did not directly assess knowledge; instead, it assessed confidence in knowledge. Confidence has greater subjectivity than knowledge-based assessments and is not a reliable alternative to assessing student learning. Therefore, future evaluations of these animations would benefit from a validated assessment of students before and after the tutorial.

The teaching session itself would have benefited from greater interactivity, which has been showed to be an important element to teaching [14-16]. The session was delivered in a more didactic way, compared to CBL or team-based learning methods, which may have compromised student engagement and therefore learning. None of these elements were incorporated in the teaching session mainly due to time constraints but also to maintain the focus of the session on evaluating the value of the animations in improving student confidence. For instance, the inclusion of cases would act as a confounding variable as students may be able to understand the pathology from the case rather than from interpreting the ECG, whereas other students might not have engaged as much with the animations when in a team compared to when working individually. It is important to note that clinical context is important, as supplementing teaching materials with a patient case helps students to better diagnose, investigate, and manage cardiac conditions, thus improving their clinical reasoning skills [16].

The animations are likely to be even more valuable if used alongside other helpful learning tools, including the design of more interactive tutorials by involving quizzes throughout and gamification, which is a concept recently discussed in the literature, wherein game design elements are used in nongame contexts to promote users’ engagement [17]. Moreover, future teaching sessions would benefit from including the aforementioned clinical scenarios prior to demonstrating each pathology, intertwining the learning of relevant pathophysiology with clinical knowledge. Although the latter would allow greater contextualization and demonstrate the relevance of the learning to clinical practice, the former would provide the required background knowledge to understand the clinical manifestations, and management, of disease.

In addition, it is important to acknowledge that for students to confidently be able to interpret ECGs, they need to apply the concepts of spaced learning and repetition. Future teaching could be accompanied by resources such as a recording of the session, the slides and animations used, as well as single–best answer questions to enable students to consolidate and test their learning. A more appropriate method of using these animations may therefore be to provide these to students as an independent learning resource. When doing so, it would enable students to scroll through the animation and independently control its speed to match their learning needs and understanding. Additionally, students suggested to make the animation 3D and to demonstrate the full 12-lead ECG alongside the animation as opposed to a single lead only.

In conclusion, this study suggests that although incorporating visual animations to demonstrate the electrical activity of different pathologies in ECG teaching may be beneficial in improving students’ confidence in interpreting ECGs and understanding the underlying pathology, it is not the only way that this can be achieved. Students benefited equally from verbal explanations, suggesting that the most essential part of future ECG teaching is providing emphasis on the relevant pathophysiology, presented alongside clinical vignettes in which discussions regarding investigations and management options can be made. Interactivity within teaching sessions using quizzes and spaced practice is also recommended, in which students can access the resources, including the animation used in the session, later, to help consolidate their learning. Nevertheless, the development of animations was a low-cost intervention enjoyed by students and was reported to support their learning and understanding of cardiac pathophysiology and interpretation of ECG traces. Therefore, it is hoped that making these animations available to students as a revision resource can supplement their current ECG teaching and individual study practices.

## Supplementary material

10.2196/46507 Multimedia Appendix 1 Questionnaires.

10.2196/46507 Multimedia Appendix 2 Focus group questions.

10.2196/46507 Multimedia Appendix 3 Intervention data.

10.2196/46507 Multimedia Appendix 4 Control data and figures.

10.2196/46507 Multimedia Appendix 5 Raw data.

## Abbreviations

AF: atrial fibrillation
BBB: bundle branch block
CBL: case-based learning
ECG: electrocardiography
