# Supplementary material for: The Use of Animations Depicting Cardiac Electrical Activity to Improve Confidence in Understanding of Cardiac Pathology and Electrocardiography Traces Among Final-Year Medical Students: Nonrandomized Controlled Trial
Source: JMIR Med Educ. 2024 Apr 23;10:e46507. doi: 10.2196/46507 (PMC11063581; doi:10.2196/46507)
Supplement: Multimedia Appendix 1 [file mededu-v10-e46507-s001.docx]

**Pre-Intervention Questionnaire**

**Study title:** The impact of simultaneous anatomical and electrical animations on interpreting and understanding ECGs amongst third year medical students.

Please state your unique identifier code (this was emailed to you):

1. I confirm that I have read, understood and agree with the details outlined in the participant information sheet (Yes)
2. What type of ECG teaching have you received in the past? (Tick all that apply)

- Didactic Lectures
- Case-Based Tutorials
- Animation
- Practicals
- Rote-Learning ECG Features
- Other: (open)

1. How confidently can you review an ECG and diagnose the following rhythms (1: Not confidently at all; 5: Very confidently):
   1. Sinus rhythm
   2. Atrial flutter
   3. Atrial fibrillation
   4. AVNRT
   5. AVRT
   6. Right bundle branch block
   7. Left bundle branch block
2. How confidently can you visualise the passage of electrical activity through the heart in the following rhythms (1: Not confidently at all; 5: Very confidently):
   1. Sinus rhythm
   2. Atrial flutter
   3. Atrial fibrillation
   4. AVNRT
   5. AVRT
   6. Right bundle branch block
   7. Left bundle branch block
3. Overall, how confident do you feel about your ability to interpret ECGs as a junior doctor when you start working? (1: Not confident at all; 5: Very Confident)
4. How have you enjoyed ECG teaching in the past? (1: not enjoyed at all; 5: enjoyed it very much)
5. What aspects of your past ECG teaching do you think could be improved, generally? Please explain (Free Text)

**Post-Intervention Questionnaire**

**Study title:** The impact of simultaneous anatomical and electrical animations on interpreting and understanding ECGs amongst third year medical students.

Please state your Unique Identifier:

1. How confidently can you review an ECG and diagnose the following rhythms (1: Not confidently at all; 5: Very confidently):
   1. Sinus rhythm
   2. Atrial flutter
   3. Atrial fibrillation
   4. AVNRT
   5. AVRT
   6. Right bundle branch block
   7. Left bundle branch block
2. How confidently can you visualise the passage of electrical activity through the heart in the following rhythms (1: Not confidently at all; 5: Very confidently):
   1. Sinus rhythm
   2. Atrial flutter
   3. Atrial fibrillation
   4. AVNRT
   5. AVRT
   6. Right bundle branch block
   7. Left bundle branch block
3. Overall, how confident do you feel about your ability to interpret ECGs as a junior doctor when you start working? (1: Not confident at all; 5: Very Confident)
4. What did you think about the clarity of the animations used? (1: Extremely unclear, 5: Extremely Clear)
5. Overall, how much did you enjoy the session? (1: not enjoyed at all; 5: enjoyed it very much)
6. What did you find most helpful or beneficial in this tutorial? [Free text]
7. What do you think should be improved in this tutorial? [Free text]
