# Supplementary material for: The Use of Animations Depicting Cardiac Electrical Activity to Improve Confidence in Understanding of Cardiac Pathology and Electrocardiography Traces Among Final-Year Medical Students: Nonrandomized Controlled Trial
Source: JMIR Med Educ. 2024 Apr 23;10:e46507. doi: 10.2196/46507 (PMC11063581; doi:10.2196/46507)
Supplement: Multimedia Appendix 2 [file mededu-v10-e46507-s002.docx]

**Focus Group Questions**

1. How have you been taught ECGs in the past?
   1. What was your experience with this method of teaching?
   2. What did you enjoy about your past ECG teaching?
   3. What did you find challenging / what would you like to see improved about your past ECG teaching?
2. Overall, how do you find learning about ECGs in the past?
   1. Which aspect(s) of ECG interpretation did you find most difficult before the tutorial that we delivered?
3. How did you find that the tutorial affected your understanding of ECG (waveforms and ECG interpretation)?
   1. What was it about the tutorial that helped improve your understanding of ECGs?
   2. How was your experience with the animations depicting electroanatomical and electrocardiographic? How did they affect your understanding of ECGs?
4. What did you find beneficial about the tutorial?
5. How could the tutorial be improved to add further benefit to your understanding of ECGs?
   1. How could the animation be improved to add further benefit to your understanding of ECGs?
6. How did your experience in this tutorial compare to previous experiences of ECG teaching?
