# Supplementary material for: The Use of Animations Depicting Cardiac Electrical Activity to Improve Confidence in Understanding of Cardiac Pathology and Electrocardiography Traces Among Final-Year Medical Students: Nonrandomized Controlled Trial
Source: JMIR Med Educ. 2024 Apr 23;10:e46507. doi: 10.2196/46507 (PMC11063581; doi:10.2196/46507)
Supplement: Multimedia Appendix 4 [file mededu-v10-e46507-s004.docx]

**Multimedia Appendix 4. Control Group: Data and Figures**

**Table S1** – Median confidence scores, IQR and Wilcoxon Signed-Rank test results for confidence reviewing ECGs and diagnosing rhythms pre- and post-tutorial for specified cardiac pathologies (n=13; control group)

|  | | **Sinus rhythm** | | **Atrial flutter** | | **Atrial fibrillation** | | **AVNRT** | | **AVRT** | | **RBBB** | | **LBBB** | |
| --- | --- | --- | --- | --- | --- | --- | --- | --- | --- | --- | --- | --- | --- | --- | --- |
|  | | ***Pre*** | ***Post*** | ***Pre*** | ***Post*** | ***Pre*** | ***Post*** | ***Pre*** | ***Post*** | ***Pre*** | ***Post*** | ***Pre*** | ***Post*** | ***Pre*** | ***Post*** |
| **Median score** | | 5 | 5 | 3.3 | 4.3 | 4 | 4.5 | 2 | 3.5 | 2 | 3.7 | 2.5 | 3.9 | 2.5 | 4.3 |
| **IQR** | | 1.1 | 0.8 | 1 | 1.6 | 0.9 | 1.3 | 1.2 | 1.4 | 1 | 1.2 | 1.5 | 1.5 | 1.5 | 1.5 |
|  | |  | |  | |  | |  | |  | |  | |  | |
| **Wilcoxon Signed-Rank Test** | ***N-value*** | 5 | | 11 | | 12 | | 11 | | 12 | | 11 | | 12 | |
|  | **W-value** | Can’t determine | | 2.5 | | 22.5 | | 0 | | 0 | | 0 | | 1 | |
|  | **Significance**  *** *P* *≤*.05**  **** *P≤*.01**  ***** *P≤*.001** | N/A | | ** | | Not significant | | *** | | *** | | *** | | *** | |

**Table S2** – Median confidence scores, IQR and Wilcoxon Signed-Rank test results for visualising cardiac electrical activity pre- and post-tutorial for specified cardiac pathologies (n=13; control group)

|  | | **Sinus rhythm** | | **Atrial flutter** | | **Atrial fibrillation** | | **AVNRT** | | **AVRT** | | **RBBB** | | **LBBB** | |
| --- | --- | --- | --- | --- | --- | --- | --- | --- | --- | --- | --- | --- | --- | --- | --- |
|  | | ***Pre*** | ***Post*** | ***Pre*** | ***Post*** | ***Pre*** | ***Post*** | ***Pre*** | ***Post*** | ***Pre*** | ***Post*** | ***Pre*** | ***Post*** | ***Pre*** | ***Post*** |
| **Median score** | | 4 | 5 | 2 | 5 | 2 | 5 | 2 | 4 | 2 | 4 | 2 | 4 | 2 | 4 |
| **IQR** | | 2 | 1 | 1 | 1 | 3 | 1 | 2 | 2 | 1 | 2 | 3 | 1 | 3 | 1 |
|  | |  | |  | |  | |  | |  | |  | |  | |
| **Wilcoxon Signed-Rank Test** | ***N-value*** | 9 | | 10 | | 10 | | 12 | | 12 | | 10 | | 10 | |
|  | **W-value** | 3 | | 0 | | 0 | | 0 | | 0 | | 0 | | 0 | |
|  | **Significance**  *** *P* *≤*.05**  **** *P≤*.01**  ***** *P≤*.001** | ******* | | ** | | ** | | *** | | *** | | ** | | ** | |


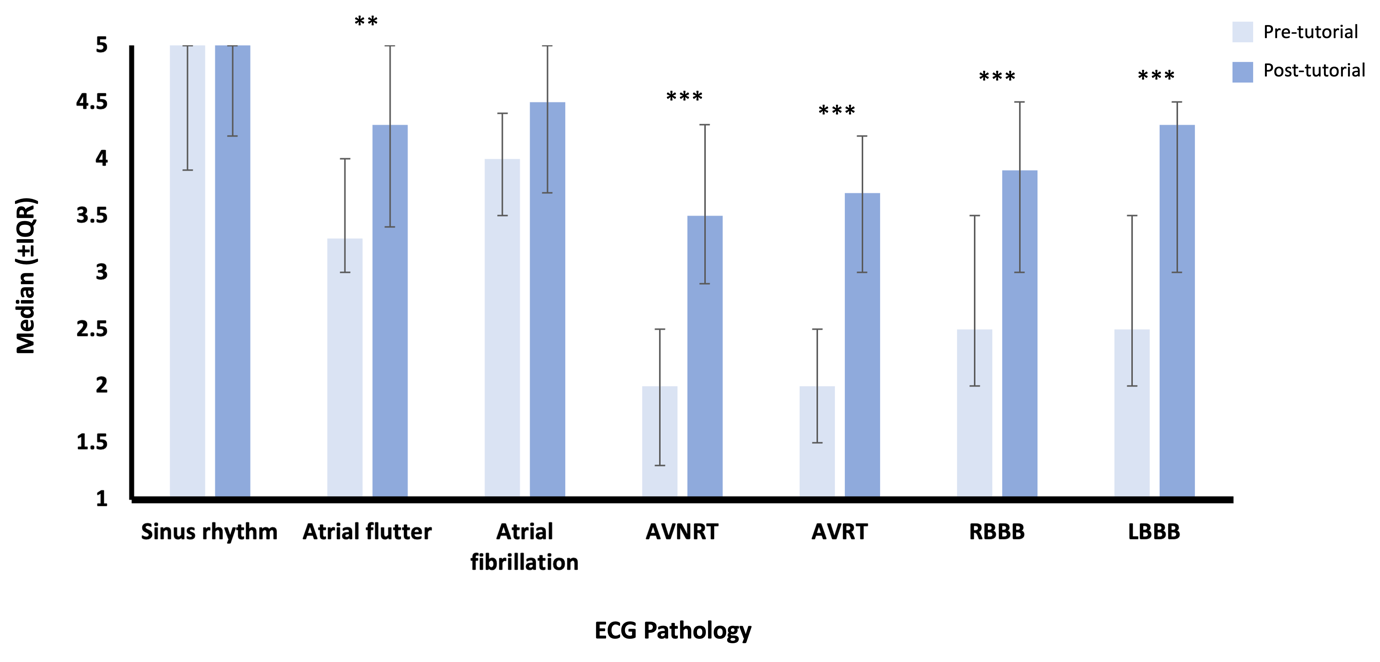


**Figure S1** - Confidence reviewing and diagnosing cardiac rhythms and pathology on an ECG (median score from a 1 (not confident at all) to 5 (extremely confident) Likert scale ± interquartile range); Control group (n=13)


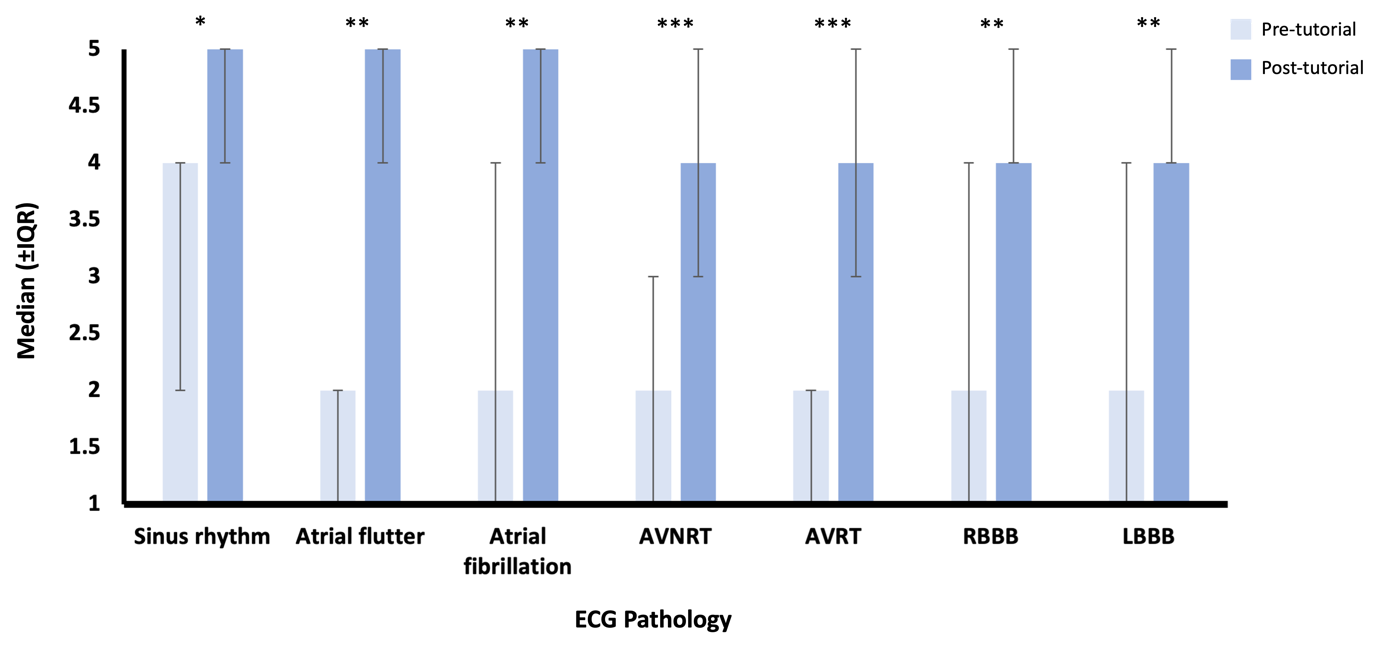


**Figure S2** - Confidence visualising electrical activity through the heart in different ECG pathologies (median score from a 1 (not confident at all) to 5 (extremely confident) Likert scale ± interquartile range); Control group (n=13)
